# Invasive Treatment of Left Main Coronary Artery Disease: From Anatomical Features to Mechanistic Differences

**DOI:** 10.2174/011573403X321064240715061250

**Published:** 2024-07-15

**Authors:** Hristo Kirov, Tulio Caldonazo, Torsten Doenst

**Affiliations:** 1 Department of Cardiothoracic Surgery, Jena University Hospital, Friedrich Schiller University of Jena, Jena, Germany

**Keywords:** Coronary artery bypass grafting, percutaneous coronary intervention, coronary artery disease, LM stenosis, LM shaft, anatomical features

## Abstract

There is debate on the best treatment for significant stenoses of the left main (LM) coronary artery. The available evidence is based on four randomized trials, which were either performed specifically to assess patients with LM disease (EXCEL, NOBLE, PRECOMBAT) or had a significant fraction of patients with this disease pattern (SYNTAX). A meta-analysis revealed no difference in periprocedural and 5-year mortality but demonstrated a significant reduction of spontaneous myocardial infarction (MI) with CABG. Furthermore, the recently published SWEDEHEART registry data have shown survival advantage and fewer MACCE with CABG for LM disease after adjustment. In general, patients with more severe coronary artery disease (CAD) appear to have a survival advantage with CABG both over PCI and medical therapy (independent of the presence or absence of LM stenosis), which is always associated with a reduction of spontaneous MI in the CABG arm. Since the nomenclature of LM disease does not automatically reflect the complexity of CAD, we review the nature of LM disease in this article. We mechanistically assess the treatment effects of PCI and CABG for patients with LM disease, which is rarely isolated, often distal, and mostly associated with varying degrees of single and multi-vessel disease. We conclude that in patients with isolated LM shaft lesions and associated diseases of low complexity, the risk of spontaneous MI is lower, and PCI may achieve similar long-term outcomes compared to CABG. Thus, heart teams are essential for selecting the best treatment option and should focus on assessing infarction risk in chronic CAD.

## 1 INTRODUCTION

Heated debates have arisen regarding the best invasive treatment option for significant stenoses of the left main coronary artery [1]. Early surgical studies demonstrated a survival advantage of bypass grafting over medical therapy [2]. Since different comparisons of CABG with percutaneous coronary intervention (PCI) have illustrated non-inferiority of PCI under selected conditions (generally less complex coronary disease), the inference was made that PCI is also able to improve life expectancy under these conditions. This reasoning is the basis for the current guideline recommendations on the treatment of patients with left main stenosis and decision-making [3, 4].

Guideline recommendations, in general, are primarily based on the evaluation of trial outcomes (ideally randomized), and the results from selected and small groups of patients (considering the millions of people affected by CAD) are used to deduct recommendations for all. It is clear that many factors can, therefore, affect the outcomes of even the best-randomized trials. Such factors may pertain to primary endpoint selection and composition which define endpoints(take the definition of postoperative myocardial infarction in EXCEL) as a prominent example (Society for Cardiovascular Angiography and Interventions (SCAI) definition: creatinine kinase-MB (CK-MB) cut-off of >10x URL for both PCI and CABG (or troponin >70x URL); no inclusion or exclusion criteria was required [5, 6]. Thus, the following question arises, “should we put the results into perspective for the often very heterogeneous presentation of the disease”?

The statistical significance testing assesses the chance to wrongfully reject or accept an assumption made with a hypothesis, but does not necessarily uncover the truth. Thus, a comparison of groups with different characteristics (*e.g.*, patients having received CABG or PCI) requires a plausible hypothesis for an underlying mechanism. Otherwise, we may have to accept the significant association between the number of storks and the birth rate of babies [7] as proof that the stork actually delivers them.

Two key questions remain in the context of CABG and PCI comparisons for the patients with Left Main Disease. First, what do we actually mean by left main diseasefor patients having issues in the non-left main coronary areas (*i.e.*, single, double, and triple vessel disease). Second, what is the reason (*i.e.*, the difference in treatment mechanisms) for observed differences between CABG and PCI in patients with left main disease? We here review the field with a focus on these two questions.

## 2 WHAT IS THE LEFT MAIN DISEASE?

Clinical trials and the guidelines always refer to patients with left main CAD as a subcategory to patients with single double or triple vessel disease. This subcategorization may lead to the understanding that patients with left main disease are different from those with non-left main affection of the coronary arteries. A recent analysis from the SWEDEHEART registry addressed this question [8]. The authors analyzed 2.6 million coronary artery segments from coronary angiograms of almost 250,000 patients. They selected patients who received repeated diagnostic angiograms and identified over 26,000 coronary artery segments that progressed to obstructive CAD. Two-thirds of these obstructive lesions were invasively treated either with PCI (56.4%) or with CABG (4.9%). Fig. (**1**) illustrates the distribution of the obstructive disease in this patient population. The left main coronary artery was affected in only 5% of patients. The most frequently affected vessel was the left anterior descending artery (LAD). In addition, the progression of coronary artery disease within a 15-year observation time period was the fastest in the LAD region and the slowest in the left main, suggesting that the left main disease may be more stable, compared to, LAD disease.

Reviewing other investigations assessing the incidence and relevance of left main disease suggests that isolated left main disease is a rare commodity. D’Allones *et al*. [9] found that surgery for the isolated left main disease reflected only 1.4% of all CABG procedures in the analyzed time period. An epidemiological survey of 15,000 patients undergoing coronary angiograms in a single tertiary cardiac hospital in India showed significant left main disease in 2.2% and isolated left main ostial disease in only 0.18% [10]. The authors additionally identified younger age and female sex as associated factors with isolated left main disease.

At the same time, the SWEDEHEART analysis found the left main to be affected in only 5% of the cases but showed that the presence of left main affection is associated with additional multivessel disease in almost 2/3 of those patients [8].

In summary, isolated left main coronary artery disease appears to be a rare commodity, affecting less than 5% of all patients with CAD. It may even be different from classic coronary artery disease because more women and younger patients seem to be affected [10]. Currently, the most frequent presentation of left main disease is in association with the presence of multivessel coronary artery disease [8].

## 3 CURRENT EVIDENCE FOR LEFT MAIN DISEASE

The currently available randomized evidence is based on four trials which were either performed specifically to assess patients with the left main disease (EXCEL [11], NOBLE [12], and PRECOMBAT) [13] or had a significant fraction of patients with this disease pattern (SYNTAX [14]). The trials specifically devoted to patients with left main disease reported outcomes that were somehow contradictory, and specifically, the EXCEL trial created a massive controversy in the community by showing results modified by the classification of periprocedural MI used Fig. (**2A**). While NOBLE demonstrated no difference in survival, the primary composite end-point consisting of death, myocardial infarction, stroke, and the need for re-revascularization was different between the groups with the conclusion that PCI did not meet the non-inferiority criteria compared to CABG (28% - 165 events for PCI and 19% - 110 events for CABG, HR 1·58 [95% CI 1·24–2·01]) Fig. (**2B**). The primary outcome was mainly driven by a significantly lower rate of spontaneous myocardial infarctions in the CABG group. Periprocedural infarctions were excluded in this trial. In contrast, the EXCEL trial introduced a new definition for periprocedural myocardial infarction [6], which allowed the identification of myocardial infarctions by isolated biomarker elevations only (*i.e.*, CK-MB release). Furthermore, because of this unique definition for myocardial infarction (a 10-fold elevation of CK-MB level alone qualified as MI), the composite end point of death, stroke, or myocardial infarction at three years was not significantly different, although there was an absolute 3% lower all-cause mortality in the CABG group. The conclusion of PCI being non-inferior to CABG sparked controversy, which reached its peak with documentaries on public international news media [15].

Additionally, to calm the waves, the independent TIMI group conducted an individual patient-data meta-analysis of all four trials with relevant numbers of left main patients [16]. Figs. (**2C** and **D**) show the main outcomes of this analysis, which revealed no difference in periprocedural and 5-year all-cause mortality but demonstrated a significant reduction of spontaneous myocardial infarction with CABG.

Randomized data are often criticized for their high level of selectivity. In contrast, registry data may be considered to reflect the outcomes for a large fraction of the affected patient population and may, therefore, serve as external validation of the RCTs outcomes, although they are often heavily burdened with various biases. Nevertheless, a recent all-comers prospective registry study (the SWEDEHEART registry [17]) in patients with left main CAD showed that PCI was associated with higher mortality and higher incidence of major adverse cardiovascular and cerebrovascular events (MACCE; death, MI, stroke, or new revascularization) than CABG after adjustments for known cofounders. Importantly, the study also showed a quantitative interaction between diabetic status and mortality, translating into a median survival time of 3.6 years longer, favoring CABG in patients with diabetes. In the entire cohort, myocardial infarction occurred in 9.3% of CABG and in 15.7% of PCI patients. Further evidence supporting the better long-term outcomes with CABG comes from a registry analysis analyzing outcomes in Ontario, Canada, where CABG outperformed PCI in the long run (7 years follow-up) in terms of mortality, myocardial infarction and re-revascularization at similar 30-day mortality but higher periprocedural stroke rates [18].

**Fig. (3):**
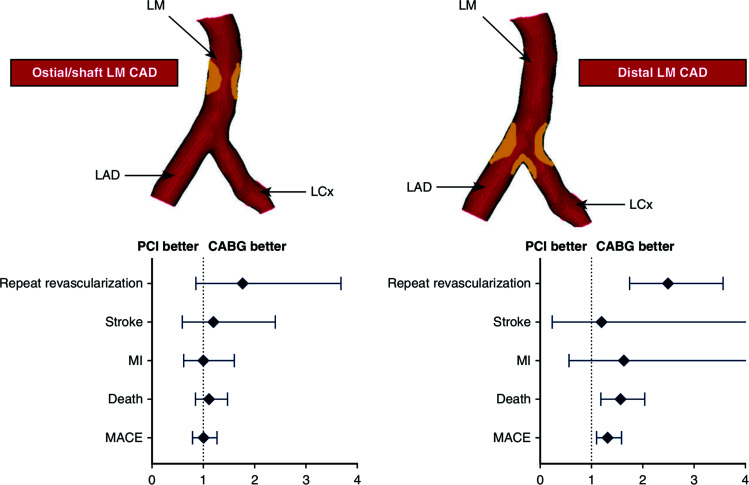
Central Illustration from a meta-analysis comparing CABG and PCI in left main disease focusing on the site of the left main lesion (19) (reproduced with permission). CAD: Coronary Artery Disease, LAD: Left Anterior Descending Artery, LCx: Left Circumflex Artery, MACE: Major Adverse Cardiovascular Events, MI: Myocardial Infarction.

Finally, a classic meta-analysis assessed the role of the localization of the left main disease on outcomes based on the invasive treatment modality chosen (Fig. **3**) [19]. While there was no difference between CABG and PCI if the left main disease was in the ostium or the shaft, there were significantly more events, including death in the PCI group, when the distal left main, including the bifurcation, was affected. Davidson *et al*. addressed the complexity of LM disease in a state-of-the-art review [20] and suggested an algorithm for daily action. In addition to this elegant summary, it is important to realize that CABG in the LM trials was associated with reduced numbers of myocardial infarctions.

The pattern of less myocardial infarctions (fatal or non-fatal) in CABG groups is not new and always emerges when coronary artery disease is complex (*i.e.*, the risk of myocardial infarction is high) [21-23]. Thus, if the risk of an event from any coronary lesion is high, CABG appears superior, which raises the question of the underlying mechanism to explain this difference.

Furthermore, there is still an ongoing debate as to whether periprocedural myocardial infarction represents a relevant clinical event or just a biochemical/laboratory finding that does not correlate with a change in the patient's prognosis. Analyses show that it does not correlate with mortality and possibly not with quality of life in the long run [24]. The relationship between the survival advantage of CABG and its ability to prevent myocardial infarctions involves different mechanisms compared to PCI.

Patients with coronary artery disease either present with symptoms upon exertion or with an acute coronary event (acute coronary syndrome and/or myocardial infarction). The main symptoms are angina pectoris or dyspnea, which can be linked to atherosclerotic coronary plaques obstructing flow and cause ischemia if the demand for blood supply increases and flow capability reaches its limits. This type of ischemia has been termed inducible ischemia and is reversible once exertion is terminated. In patients with ischemia at rest, obstructions and flow limitations have reached a stage where coronary flow at rest is insufficient, and under these conditions, myocardium is in immediate jeopardy. Under these acute conditions, immediate treatment is required in order to limit or prevent muscle loss. In contrast, the treatment of chronic coronary syndrome (defined as coronary disease in the absence of acute/ongoing ischemia at rest) pursues the goal of treating symptoms from inducible ischemia and attempts to avoid the transition to acute coronary syndrome.

Assessing the literature from this perspective illustrates that both PCI and CABG are equally able to reduce the onset of symptoms at exertion and improve quality of life [25, 26].

The question of whether invasive treatment of CAD prolongs life is more than four decades old [27], but the underlying effective mechanism has never been addressed specifically. It was always assumed that “revascularization” is responsible, although several findings from randomized and non-randomized evidence argue against the modulation of the amount of blood supply being responsible for any prognostic treatment effect.

**Fig. (4):**
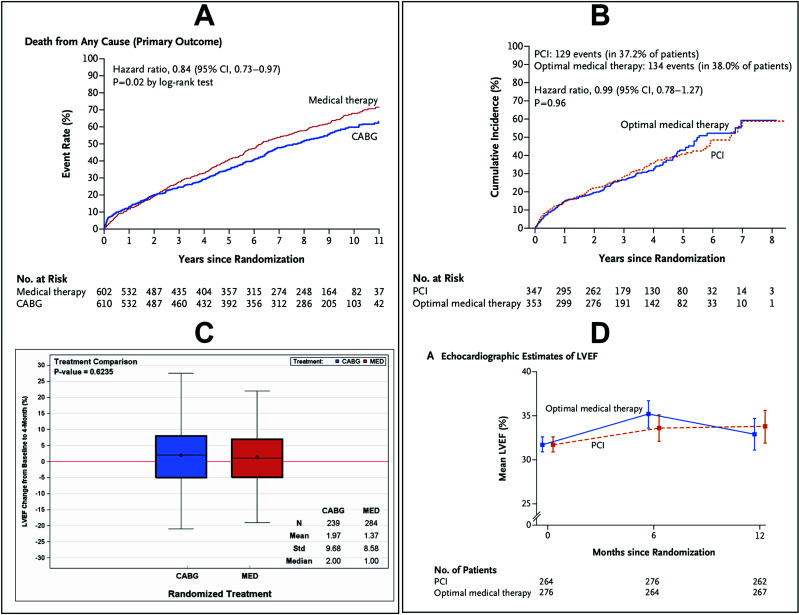
**A:** All-cause mortality in the STICH trial (29) (reproduced with permission). **B:** All-cause mortality or heart failure rehospitalization in the REVIVED BCIS-2 trial (30) (reproduced with permission). **C:** Change in EF from Baseline to 4 months after treatment with CABG or medical therapy in the STICH trial (51) (reproduced with permission). **D:** EF over time with either PCI or medical treatment in the REVIVED BCIS-2 trial (30) (reproduced with permission). CI: Confidence Interval, HR: Hazard Ratio, LVEF: Left Ventricular Ejection Fraction, MED: Medical Therapy.

A recent meta-analysis reconfirmed the life-prolonging effect of CABG over contemporary medical therapy [28]. The only randomized trial that demonstrated a survival benefit of CABG over medical therapy alone was the STICH trial, showing an extension of 18 months of life expectancy within a ten-year observation period with CABG (Fig. **4A**) [29]. The trial included patients with significant coronary artery disease and ejection fractions below 35%. The most recently published REVIVED trial assessed the impact of PCI in a similar patient population but failed to demonstrate an impact on survival (Fig. **4B**) [30]. Thus, the successful treatment of obstructive lesions may not be the explanation for the CABG-associated survival effect. In addition, sub-studies from the STICH trial failed to associate the treatment effect with the presence or absence of viability or the degree of inducible ischemia [31, 32]. Finally, both trials failed to demonstrate an improvement in ejection fraction that was larger than with medical therapy, again questioning that modulating the amount of blood supply under these chronic conditions is responsible for the CABG-associated impact on survival (Figs. **4C** and **D**) [30, 33].

In patients with normal ejection fraction but relevant CAD, not a single trial from a whole series of studies comparing PCI to medical therapy demonstrated an impact of PCI on survival. In contrast, a patient-level meta-analysis of all prospective randomized trials comparing CABG and PCI in patients with multi-vessel disease demonstrated a significant survival advantage of CABG after five years compared to PCI [34]. These findings from randomized data were validated by a systemic review of the literature assessing all registry data comparing CABG and PCI in clearly identified regions of this world [35]. In this analysis, CABG provided a 5 to 6% total survival advantage over PCI, irrespective of the region where invasive treatment was conducted. Thus, the evidence for a superior treatment effect of CABG over PCI, specifically in complex coronary artery disease, appears to be a global and universal finding that cannot be explained by modulations of blood supply addressed in these chronic situations.

**Fig. (5):**
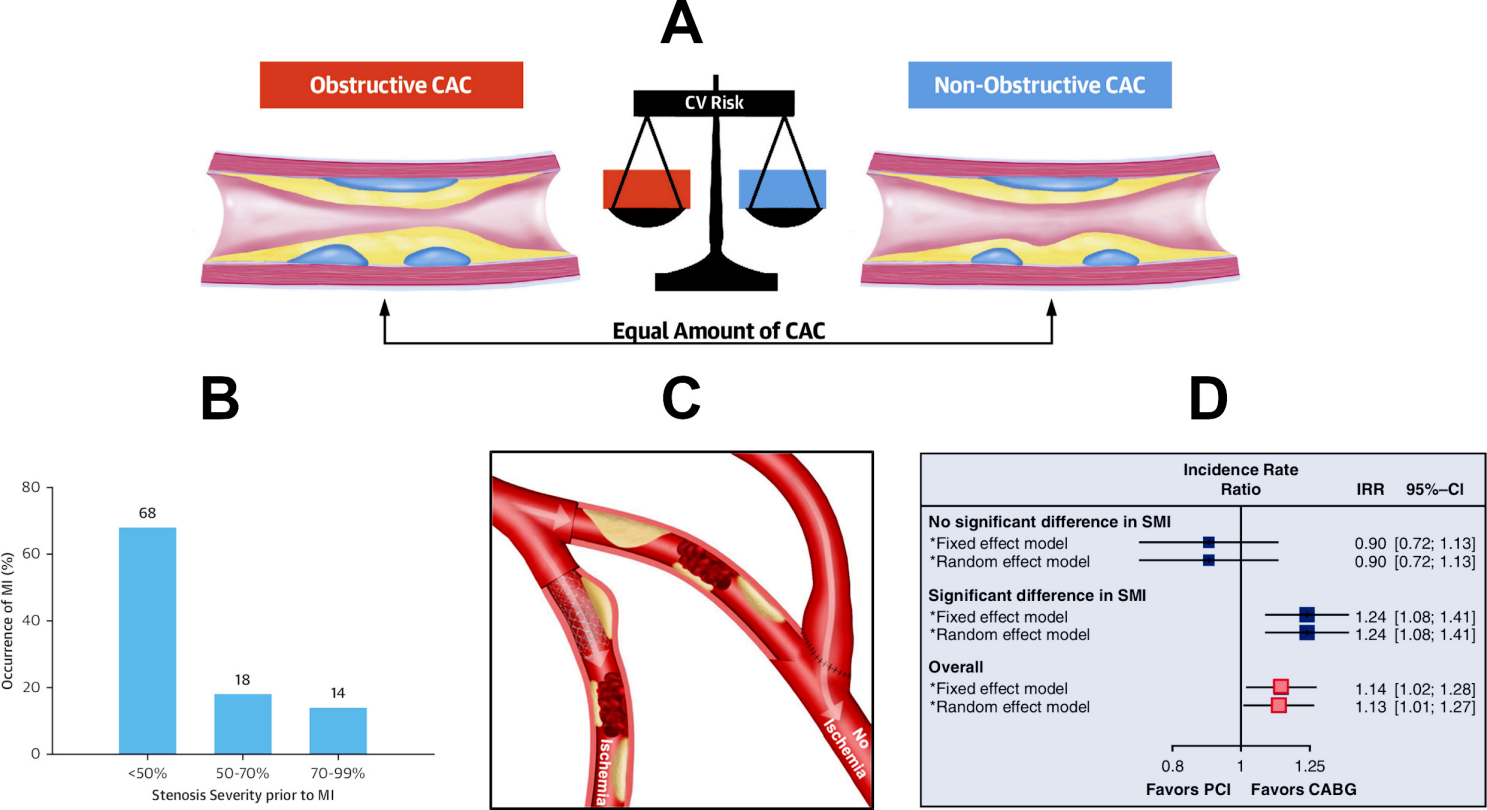
**A:** Comparison of cardiovascular risk from obstructive and non-obstructive coronary artery disease from CT calcium scoring studies (36) (reproduced with permission). **B:** Quantification of myocardial infarction occurrence as a function of stenosis severity of the underlying atherosclerotic lesion (21) (reproduced with permission). **C:** illustration of the mechanistic principle of surgical collateralization (21) (reproduced with permission). **D:** Relationship between a CABG-associated survival advantage and its ability to prevent myocardial infarctions (39) (reproduced with permission). CAC: Coronary Artery Calcium, CI: Confidence Interval, CV: Cardiovascular, IRR: Incidence Rate Ratio, MI: Myocardial Infarction, SMI: Spontaneous Myocardial Infarction.

Furthermore, searching for an alternative explanation for the underlying mechanisms, it is striking to note that the risk of suffering a coronary event appears to be independent of the degree of obstructiveness in a given coronary artery disease setting. Analyses of coronary CT data assessing obstructive and non-obstructive CAD based on calcium scoring [36] clearly illustrate that the degree of coronary obstruction may not increase the risk of suffering from a coronary event (Fig. **5A**). Previous evidence from anatomical studies have already suggested that the vast majority of myocardial infarctions arise from coronary atherosclerotic lesions that are not flow-limiting (Fig. **5B**) [21,37]. Thus, addressing only flow-limiting lesions by PCI may not be able to provide protection from future myocardial infarctions [21, 23, 38]. In contrast, CABG, where grafts are generally placed distal to the entire atherosclerotic disease, provides surgical collateral that does not only improve blood supply at exertion for treatment of an obstructive lesion but also prevents the development of future myocardial infarctions arising from plaque rupture in the coronary vessel proximal to bypass graft insertion. We illustrated this concept of surgical collateralization in a recent review article [21] (Fig. **5C**) and subsequently demonstrated a striking correlation of CABGs ability to prevent spontaneous myocardial infarctions and long-term survival (Fig. **5D**) [22, 23, 39]. Furthermore, it is important to notice that non-invasive imaging modalities such as coronary CT [40] or more complex invasive procedures such as intravascular ultrasound or optical coherence tomography may allow the detection of vulnerable coronary atherosclerotic lesions, while other non-invasive imaging modalities such as exercise stress echocardiography by an expert echocardiographer may be useful in the clinical practice to identify coronary atherosclerotic lesions that are flow-limiting [41]. In addition, long-term results of CABG are related to the type of graft used and may be associated with additional molecular factors. For instance, long-lasting effects of the internal thoracic artery (ITA) have been related to the release of protective factors from the perivascular adipose tissue surrounding the ITA (*e.g.*, nitric oxide and prostacyclin) [42]”.

We further illustrated, that based on these mechanistic considerations, the term revascularization is not appropriate to describe the differential impact PCI and CABG may have in patients with coronary artery disease. Fig. (**6**) shows a summary of the different mechanisms that can be exploited by using medical therapy, PCI, or CABG for the treatment of CAD [43]. In the event of acute/ongoing ischemia, which is not reversible upon resting, the required treatment mechanism is that of reperfusion. This can be achieved either by thrombolytic therapy, PCI, or CABG. Specifically, PCI has generated tremendous success in the treatment of acute myocardial infarction [44-48]. In the setting of inducible ischemia (hallmark of chronic coronary syndrome), PCI and CABG both improve maximal coronary flow capability upon exertion, which perfectly explains symptomatic relief upon exertion in these patients. However, given the fact that the majority of myocardial infarctions arise from non-flow-limiting lesions, PCIs impact on preventing future myocardial infarctions is limited, and CABG outperforms PCI and medical therapy specifically in patients with complex coronary disease (*i.e.*, those patients with high risk of myocardial infarctions). This surgical collateralization is a CABG-specific mechanism, which appears to be complementary to the infarct preventative mechanisms of medical therapy [49].

Thus, from an anatomic and mechanistic perspective, the term “revascularization” inadequately reflects the distinct mechanisms governing the treatment effects of PCI and CABG in CAD patients also with left main disease. As commented previously, although no difference was observed between CABG and PCI when the left main disease involved the ostium or shaft, the PCI group experienced notably higher event rates, including mortality, when the distal left main, including the bifurcation, was affected. In summary, patients with more severe CAD appear to have a survival advantage with CABG both over PCI and medical therapy (independent of the presence or absence of left main stenosis), which is always associated with a reduction of spontaneous myocardial infarctions in the CABG arm. Since patients with left main disease suffer from complex multi-vessel disease in the majority of cases, CABG overall appears to outperform PCI under these conditions. Within the PCI universe, studies have shown that the single stent approach may result in lower MACE when compared to the double stent strategy for percutaneous intervention of the unprotected left main coronary artery bifurcation [50, 51].

## CONCLUSION

However, applying a mechanistic approach, differentiating acute from chronic coronary syndrome, and assessing the complexity of CAD with respect to the risk of future myocardial infarctions will better allow selecting the appropriate treatment for these patients. If the risk of myocardial infarction is high, CABG is the superior treatment option. However, if the risk of myocardial infarction is low, PCI or even medical therapy alone may be a non-inferior alternative, and the most invasive procedure (CABG) may be avoided. Lastly, we conclude that in patients with isolated LM shaft lesions and associated diseases of low complexity, the risk of spontaneous MI is lower, and PCI may achieve similar long-term outcomes compared to CABG. Thus, heart teams are essential for selecting the best treatment option and should focus on assessing infarction risk in chronic CAD.

## AUTHORS’ CONTRIBUTIONS

It is hereby acknowledged that all authors have accepted responsibility for the manuscript's content and consented to its submission. They have meticulously reviewed all results and unanimously approved the final version of the manuscript.

## Figures and Tables

**Fig. (1):**
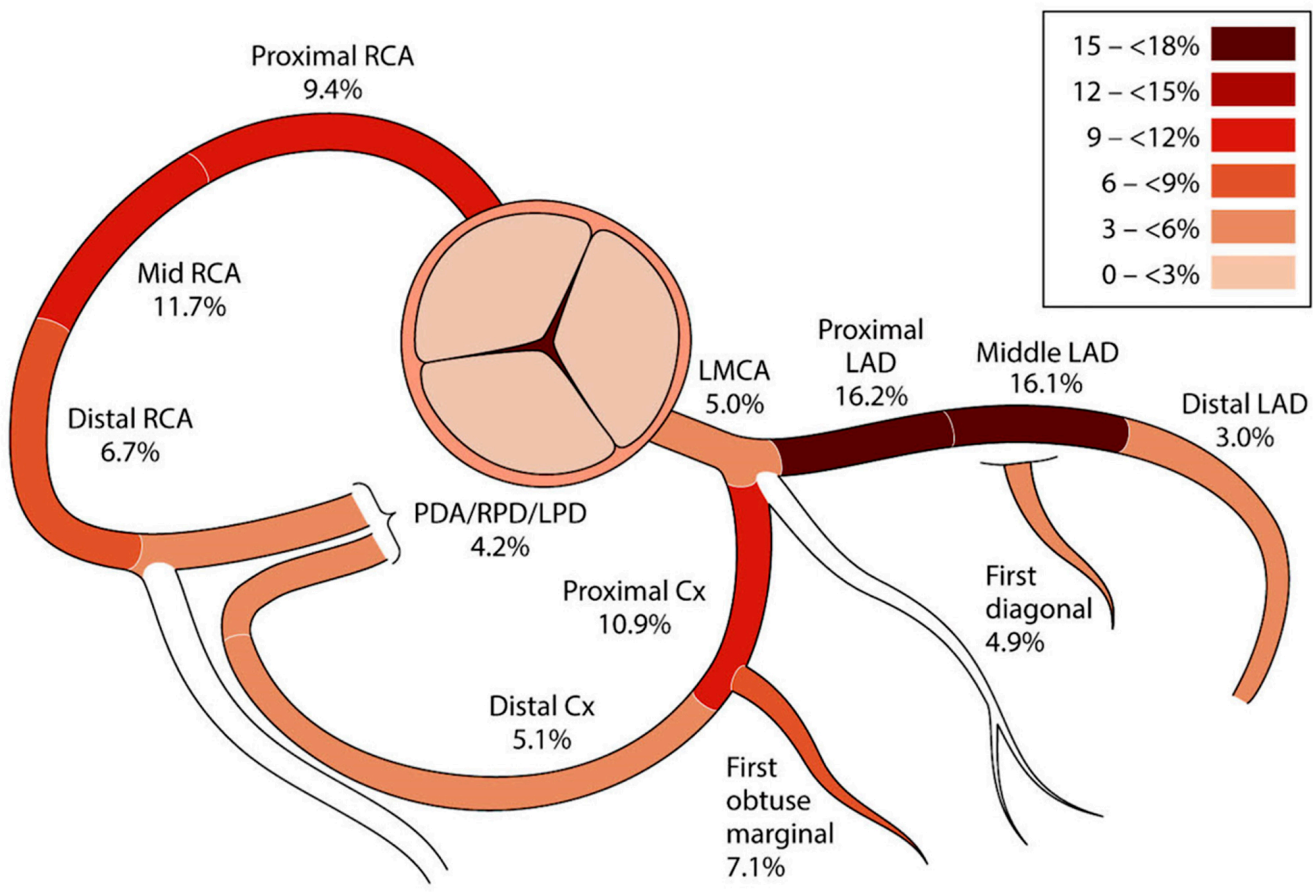
Anatomical analysis of the coronary system and its detection from obstructive coronary artery disease in 26,644 coronary segments, of which 16,298 were treated with PCI (92%) or with CABG (8%). The left main disease was present in 5% of the cases and was associated with multi-vessel affection in 60% of the cases (8) (reproduced with permission). Cx: Circumflex Artery, LAD: Left Anterior Descending Artery, LMCA: Left Main Coronary Artery, LPD: Left Posterior Descending Artery, PDA: Posterior Descending Artery, RCA: Right Coronary Artery, RPD: Right Posterior Descending Artery.

**Fig. (2):**
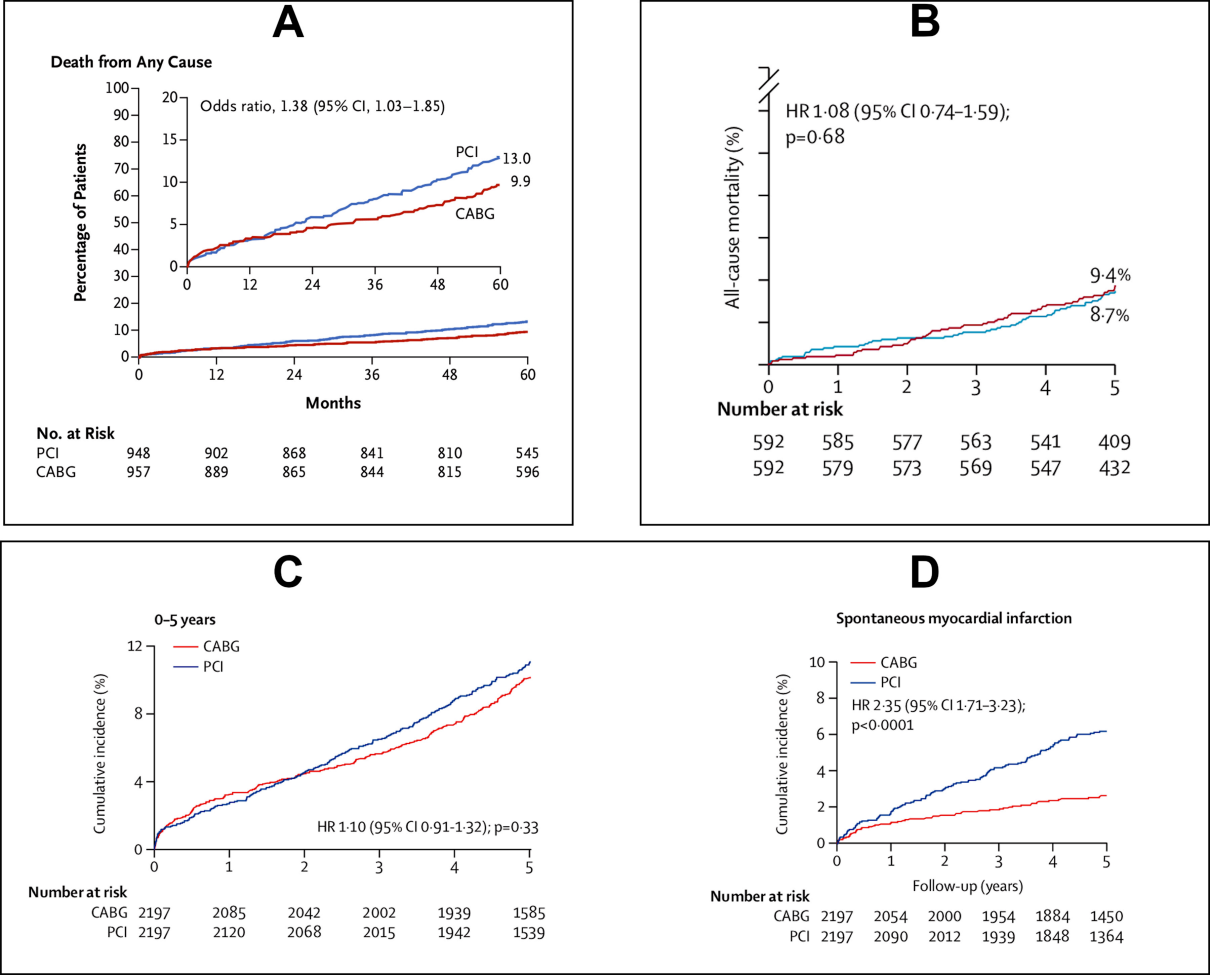
**A:** All-cause mortality in EXCEL patients (11) (reproduced with permission). **B:** All-cause mortality in NOBLE patients (12) (reproduced with permission). **C:** All-cause mortality (16) (reproduced with permission) and **D:** incidences of myocardial infractions from the patient-data meta-analysis comparing CABG and PCI in the left main disease (16) (reproduced with permission). CI: Confidence Interval, HR: Hazard Ratio.

**Fig. (6):**
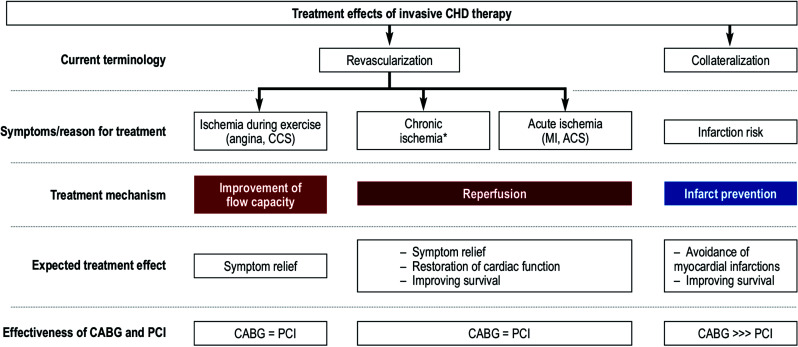
Illustration of the mechanisms underlying current invasive treatment options for coronary artery disease (43) (reproduced with permission). ACS: Acute Coronary Syndrome, CCS: Canadian Cardiovascular Society, CHD: Coronary Heart Disease, MI: Myocardial Infarction.

## LIST OF ABBREVIATIONS

LM: Left Main
MI: Myocardial Infarction
CAD: Coronary Artery Disease
PCI: Percutaneous Coronary Intervention
LAD: Left Anterior Descending Artery
ITA: Internal Thoracic Artery
